# Bidirectional ventricular tachycardia in a young woman with prominent U waves: what is the diagnosis?

**DOI:** 10.1093/ehjcr/ytag378

**Published:** 2026-05-21

**Authors:** Athanasios Saplaouras, Apostolos Theocharidis, Konstantinos P Letsas

**Affiliations:** Department of Cardiology, Onassis Cardiac Surgery Center, 356 Syngrou Avenue, Athens 17674, Greece; Private Cardiology Practice, 67 Kalvou Street, Zakynthos 29100, Greece; Department of Cardiology, Onassis Cardiac Surgery Center, 356 Syngrou Avenue, Athens 17674, Greece

## Clinical vignette

A 31-year-old woman with short stature and a broad forehead was referred to our hospital for evaluation of recurrent palpitations previously attributed to premature ventricular contractions (PVCs). She was not taking any medications.

A 12-lead electrocardiogram (ECG) showed sinus rhythm with prominent U waves (*Figure 1A*) and frequent PVCs originating from the posterior fascicle of the Purkinje system (*Figure 1B*). The corrected QT interval was within normal limits at slower heart rates, where the end of the T wave could be clearly identified. Structural heart disease was excluded on cardiac imaging, and routine blood tests were within normal ranges.

**Figure 1:**
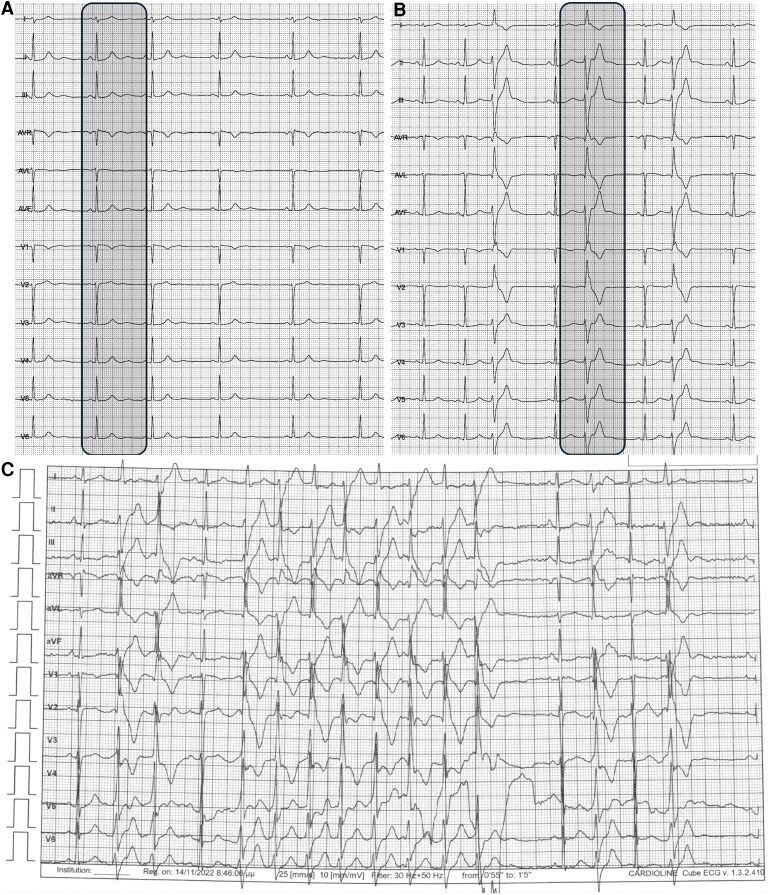
(*A*) Baseline ECG showing sinus rhythm with prominent U waves. (*B*) Premature ventricular complexes originating from the posterior fascicle. (*C*) Ambulatory ECG recording demonstrating beat-to beat alternation of the QRS axis.

An exercise treadmill test did not provoke arrhythmias. However, ambulatory ECG monitoring demonstrated several episodes of wide QRS complex tachycardia (*Figure 1C*).

## Question 1

**What is the diagnosis of this wide QRS complex tachycardia?**

- Polymorphic ventricular tachycardia
- Bidirectional ventricular tachycardia
- Supraventricular tachycardia with aberrancy
- Torsades de pointes
- Ventricular bigeminy

**Correct answer: B**

### Explanation

The tachycardia demonstrates beat-to-beat alternation of the QRS axis, which is characteristic of bidirectional ventricular tachycardia. This rare arrhythmia results from alternating ventricular activation pathways and is classically associated with conditions such as digoxin toxicity, catecholaminergic polymorphic ventricular tachycardia (CPVT), and Andersen–Tawil syndrome (ATS).^1^ Recognition of this distinctive ECG pattern is essential as it can provide an important clue to the underlying diagnosis.

## Question 2

**Considering the clinical history, baseline ECG findings, and the presence of bidirectional ventricular tachycardia, what is the most likely diagnosis?**

- Catecholaminergic polymorphic ventricular tachycardia
- Andersen–Tawil syndrome
- Digoxin toxicity
- Aconitine toxicity
- Cardiac sarcoidosis

**Correct answer: B**

### Explanation

The coexistence of dysmorphic features (short stature, broad forehead), prominent U waves, and bidirectional ventricular tachycardia strongly suggests ATS.^1–3^ Andersen–Tawil syndrome is a rare inherited channelopathy characterized by a clinical triad of periodic paralysis, ventricular arrhythmias, and characteristic skeletal or facial abnormalities. However, CPVT can also cause bidirectional ventricular tachycardia. Several features argue against this diagnosis: CPVT is not associated with dysmorphic features or prominent U waves; arrhythmias in CPVT are typically triggered by adrenergic stimulation and exercise, neither of which provoked arrhythmias in this patient; and the resting ECG in CPVT is typically normal.^1–3^

## Question 3

**Which genetic abnormality is most commonly responsible for this syndrome?**

- *SCN5A* mutation
- *RYR2* mutation
- *KCNJ2* mutation
- *LMNA* mutation
- *DSP* mutation

**Correct answer: C**

### Explanation

Genetic testing revealed a heterozygous pathogenic variant in the *KCNJ2* gene [NM_000891: c.653G>A, p.(Arg218Gln)], confirming ATS type 1. The *KCNJ2* gene encodes the Kir2.1 potassium channel, responsible for the inward rectifier potassium current (I_K1_). Loss-of-function mutations lead to abnormal ventricular repolarization, which may manifest on the ECG as prominent U waves, resulting in a predisposition to early afterdepolarizations, which are believed to initiate the bidirectional ventricular tachycardia characteristic of this condition.^1–3^

## Outcome

The patient was mildly symptomatic and was started on flecainide, resulting in a marked reduction in symptoms and arrhythmia burden during follow-up. The overall prognosis in ATS is generally favourable, with a low risk of sudden cardiac death compared with other channelopathies. Per current guidelines, implantable cardioverter-defibrillator (ICD) implantation is recommended only for prior cardiac arrest or haemodynamically unstable ventricular tachycardia (VT) and should be discussed for syncope despite medical therapy or tolerated sustained VT.^4^ As this patient met none of these criteria, ICD implantation was not pursued. Implantable loop recorder implantation was offered, but the patient declined.

## Data Availability

De-identified patient data supporting the ECG findings and clinical description are available upon reasonable request from the corresponding author.

## Lead author biography

Athanasios Saplaouras, MD, MSc, FESC, is a Consultant Cardiologist at Onassis Cardiac Surgery Center in Athens, Greece. He holds Master’s degrees in Heart Failure and Cardiomyopathies, Thrombosis and Antithrombotic Treatment, and Cardiorespiratory Resuscitation. His clinical and research interests focus on cardiac electrophysiology, ventricular arrhythmias, cardiac resynchronization therapy, and electrocardiography. He has authored numerous peer-reviewed publications and participates in clinical research studies and registries. He is also actively involved in cardiology education, resuscitation training, and scientific meetings.

## Notes

**Consent:** Written informed consent was obtained from the patient for publication of this case report and accompanying images.
